# Research progress in metabolic reprogramming and targeting metabolic pathways for clear cell renal cell carcinoma

**DOI:** 10.1016/j.gendis.2025.101991

**Published:** 2025-12-19

**Authors:** Jia-tao Yao, Peng-cheng Hu, Xiao-wei Li, Jia-cheng Xu, Ke-jie Wang, Sha-zhou Ye, Xiang-yu Meng, Hai-chao Chen, Yu Liang, Qi Ma

**Affiliations:** a Health Science Center, Ningbo University, Ningbo, Zhejiang 315211, China; b Department of Urology, No.906 Hospital of the Joint Logistic Support Force of PLA, Ningbo, Zhejiang 315040, China; c Translational Research Laboratory for Urological Diseases, The First Affiliated Hospital of Ningbo University, Ningbo, Zhejiang 315010, China; d Department of Urology, The First Affiliated Hospital of Ningbo University, Ningbo, Zhejiang 315010, China; e Comprehensive Genitourinary Cancer Center, The First Affiliated Hospital of Ningbo University, Ningbo, Zhejiang 315010, China; f Cixi Biomedical Research Institute, Wenzhou Medical University, Ningbo, Zhejiang 315300, China; g Yi-Huan Genitourinary Cancer Group, Ningbo, Zhejiang 315010, China

**Keywords:** HIFs, Metabolic reprogramming, Renal cell carcinoma, Treatment, VHL

## Abstract

Clear cell renal cell carcinoma (ccRCC), the most common pathological subtype of renal cancer, is characterized by distinctive metabolic reprogramming that serves as a core molecular pathological basis driving tumorigenesis, progression, and therapeutic resistance. The metabolic remodeling in ccRCC primarily manifests as dysregulated glucose metabolism, lipid accumulation, imbalanced amino acid utilization, and mitochondrial dysfunction. These alterations collectively sustain tumor cell bioenergetic demands, biosynthetic precursor supply, and redox homeostasis maintenance. Recent advances in multi-omics technologies and spatially resolved analytical methods have propelled ccRCC metabolism research beyond macroscopic description toward mechanistic investigation and targeted intervention. This article systematically reviews research progress focusing on metabolic reprogramming of ccRCC and the development of related therapeutic agents for this cancer.

## Introduction

Renal cell carcinoma (RCC) ranks as the third most common malignant tumor of the urinary system worldwide, accounting for 2%–3% of all adult malignancies. Its annual incidence has been steadily increasing (with an average annual growth rate of approximately 2.2%). In 2023, new cases exceeded 430,000, and deaths surpassed 179,000.^1^^,^^2^ RCC primarily includes clear cell renal cell carcinoma (ccRCC), papillary renal cell carcinoma, and chromophobe renal cell carcinoma. Among them, ccRCC is the most prevalent histological subtype, constituting 75%–80% of all kidney cancers.^3^ ccRCC exhibits distinct molecular pathological features. The core driver event in ccRCC is the inactivating mutation or epigenetic silencing of the von Hippel-Lindau (VHL) gene located on the short arm of chromosome 3 (3p), occurring in up to 90% of cases. The VHL gene encodes the VHL protein (pVHL), a critical component of the E3 ubiquitin ligase complex. The primary function of pVHL is targeted degradation of hypoxia-inducible factors (HIFs). Under normoxic conditions, HIFs are hydroxylated by prolyl hydroxylases (PHDs), recognized by pVHL and ubiquitinated, and subsequently degraded by the proteasome. Thus, HIFs only maintain low cellular levels under normoxic conditions. However, the loss of pVHL function leads to the persistent abnormal accumulation of HIFs within ccRCC cells, creating a “pseudohypoxic state”. The accumulation of HIFs results in the aberrant activation of a cascade of downstream target genes and profoundly reshapes the metabolic network of ccRCC cells, distinguishing them from normal cells.^4^ This metabolic reprogramming is a hallmark feature of ccRCC. It serves not only as a core strategy for tumor cells to adapt to the microenvironment and meet the demands of rapid proliferation (energy and biosynthetic precursors) but also as a key molecular foundation for resisting apoptosis, evading immune surveillance, promoting angiogenesis, and facilitating invasion and metastasis.^4^, ^5^, ^6^

However, this profound metabolic remodeling, driven by the VHL-HIF axis, serves not only as a cornerstone of ccRCC pathogenesis but also as a key mechanism of resistance to current standard-of-care therapies. For instance, even under oxygen-replete conditions, persistent glycolysis and massive lactate production contribute to an acidified tumor microenvironment, which inhibits the function of cytotoxic T cells and promotes M2 macrophage polarization, thereby undermining the efficacy of immune checkpoint inhibitors (e.g., anti-PD-1/PD-L1 antibodies).^7^ Similarly, the flexibility of metabolic pathways (e.g., up-regulation of lipogenesis and glutaminolysis) enables tumor cells to bypass the selective pressure imposed by tyrosine kinase inhibitors (TKIs, such as sunitinib), conferring survival advantages and leading to treatment failure through sustained energy and redox homeostasis.^8^^,^^9^ Consequently, targeting metabolic reprogramming holds immense potential not only for directly killing cancer cells but also for reversing resistance to existing therapies and creating synergistic treatment strategies. Therefore, in-depth exploration of the unique metabolic vulnerabilities of ccRCC and the development of novel therapeutic strategies targeting key metabolic nodes are urgent clinical need and have paramount scientific significance for overcoming the limitations of existing therapies and improving patient survival rates.^10^

In recent years, rapid advancements in multi-omics technologies and spatially resolved metabolic analysis methods have led to a deeper understanding of metabolic dysregulation in ccRCC. Mounting evidence suggests that targeting critical metabolic pathways, such as glucose transport, glycolytic rate-limiting enzymes, lipid synthesis and breakdown, glutamine utilization, and lactate metabolism, holds promise as a new frontier for precision therapy in ccRCC.^4^ Here, we systematically reviewed the core characteristics of metabolic reprogramming in ccRCC, focusing on its key molecular targets and mechanisms of action. We also provide a detailed assessment of the preclinical research progress and clinical trial landscape of metabolism-targeting therapeutic agents directed at these targets. The goal of this review is to offer a comprehensive reference for research and clinical translation in this field.

## Metabolic characteristics of ccRCC

Under aerobic conditions, normal cells utilize glucose for glycolysis to generate pyruvate, which is then converted to acetyl-CoA by the pyruvate dehydrogenase complex (PDC) for entry into the tricarboxylic acid (TCA) cycle. This complete oxidative process maximizes adenosine triphosphate (ATP) production through subsequent oxidative phosphorylation, providing sufficient cellular energy. Even under hypoxia, cellular sensors detect low oxygen tension and initiate adaptive responses—such as increased erythropoiesis, angiogenesis, and up-regulation of glycolytic rate-limiting enzymes’ expression and activity—enabling cells to avoid reliance on inefficient glycolysis and preferentially sustain energy production via the TCA cycle. In contrast, tumor cells exhibit a metabolic phenotype distinct from normal cells: they predominantly employ glycolysis for energy generation even in oxygen-rich environments, a phenomenon termed the Warburg effect (also known as aerobic glycolysis).^11^ This metabolic shift supports rapid tumor growth and dissemination within hypoxic and poorly vascularized microenvironments, while concurrently enhancing resistance to apoptosis and immune-mediated destruction to tumor cells.

**Figure 1:**
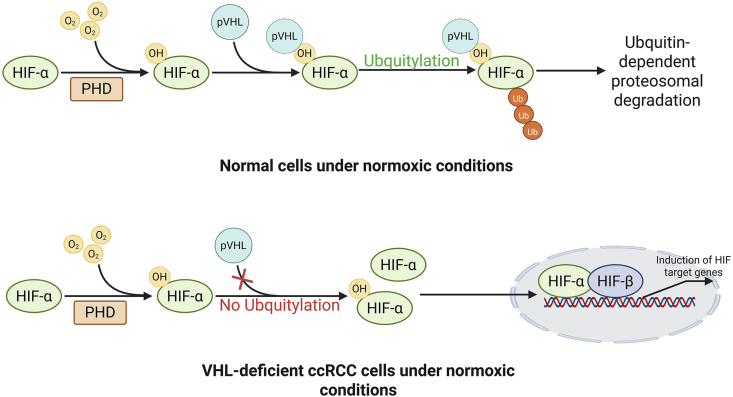
Molecular pathway of HIF-α pathological accumulation under normoxia via VHL deficiency in clear cell renal cell carcinoma. In normoxia, HIF-α is hydroxylated through catalysis by PHD enzymes. In normal cells, pVHL binds to hydroxylated HIF-α, targeting it for ubiquitination and subsequent degradation. However, in ccRCC cells, the absence of pVHL prevents the ubiquitin-mediated degradation of hydroxylated HIF-α. This results in HIF-α accumulation, nuclear translocation, and activation of hypoxia-responsive gene transcription. HIF-α, hypoxia-inducible factor α; PHD, prolyl hydroxylase-domain protein; Ub, ubiquitin; ccRCC, clear cell renal cell carcinoma; HIF-β, hypoxia-inducible factor β.

The differential responses of normal and tumor cells to hypoxia are primarily regulated by the hypoxia-inducible factor family, particularly HIF-1α and HIF-2α.^12^ These isoforms serve as primary substrates for the pVHL encoded by the *VHL* gene. HIF-1α and HIF-2α contain oxygen-dependent degradation domains (ODDDs) that mediate their interaction with pVHL. Under normoxic conditions, proline residues within ODDDs undergo iron- and oxygen-dependent hydroxylation catalyzed by PHDs. This post-translational modification enables pVHL-mediated ubiquitination and subsequent proteasomal degradation of HIFs.^12^ Consequently, HIF-α remains at minimal levels in oxygen-replete environments due to continuous proteolytic clearance. During oxygen deprivation, impaired hydroxylation leads to HIF-α accumulation. The stabilized protein dimerizes with its constitutive partner HIF-1β (aryl hydrocarbon receptor nuclear translocator), forming a functional transcription factor complex. In ccRCC, VHL mutation or inactivation disrupts pVHL-HIFs binding, preventing ubiquitination and proteasomal degradation. This creates a pseudohypoxic microenvironment characterized by constitutive HIF stabilization.^12^ Persistent HIF accumulation drives multifaceted oncogenic mechanisms, including enhanced glycolytic flux, promotion of tumor angiogenesis, and facilitation of cancer invasion and metastasis^13^ (Fig. 1).

## Key regulators of glucose metabolic reprogramming in ccRCC

**Figure 2:**
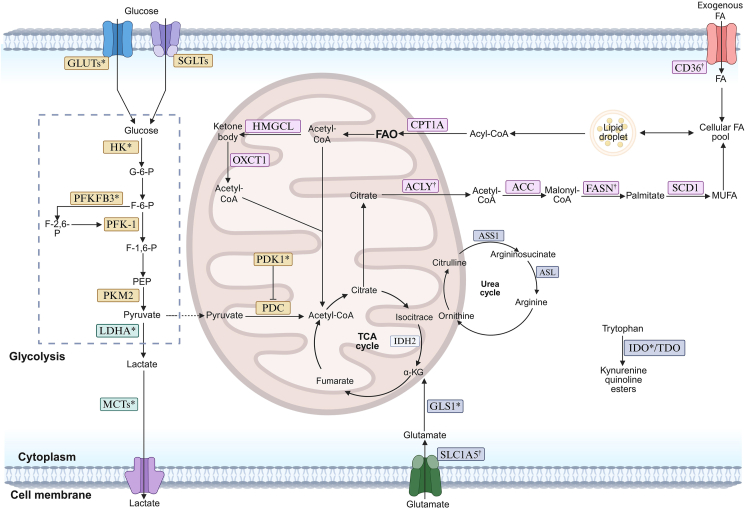
Metabolism pathways in clear cell renal cell carcinoma and potential therapeutic targets. The diagram illustrates key alterations in glucose, lipid, glutamine, and lactate metabolism driven by VHL loss and subsequent HIF activation. Molecules directly transcriptionally upregulated by HIF-1α are marked with an asterisk (∗), and those upregulated by HIF-2α are marked with a dagger (†). Key regulatory nodes and their corresponding targeted inhibitors are highlighted. ACLY, ATP-citrate lyase; ATP-citrate lyase; CPT1A, carnitine palmitoyltransferase 1 A; FA, fatty acid; FAO, fatty acid oxidation; FASN, FA synthase; F-6-P, fructose-6-phosphate; F-1,6-P, fructose-6-phosphate; GLUTs, glucose transporters; G-6-P, glucose-6-phosphate; GLS1, glutaminase 1; IDH2, isocitrate dehydrogenase 2; IDO, indoleamine 2,3-dioxygenase; LDHA, lactate dehydrogenase A; MCTs, monocarboxylate transporters; MUFA, monounsaturated fatty acid; PDC, pyruvate dehydrogenase complex; PDK1, pyruvate dehydrogenase kinase; PFK-1, phosphofructokinase-1; PFK-2, phosphofructokinase-2; PKM2, pyruvate kinase M2 isozyme; SCD1, stearoyl - CoA desaturase 1; SGLTs, sodium-glucose co-transporters; TDO, tryptophan 2,3-dioxygenase. ∗ Denotes molecules demonstrated to be direct transcriptional targets of HIF-1α (e.g., GLUT1, HK2, LDHA, PDK1, MCT4, GLS1). † Denotes molecules demonstrated to be direct transcriptional targets of HIF-2α (e.g., ACLY, CD36, SLC1A5). Molecules without symbols (e.g., CPT1A, PDC, PKM2) are regulated by indirect, post-transcriptional, or epigenetic mechanisms as detailed in the main text.

In ccRCC, the core manifestation of glucose metabolic reprogramming involves significant dysregulation of protein expression across pivotal metabolic pathways, with comprehensive investigations revealing characteristic up-regulation of key glycolytic regulators, including GLUTs, HK2, PKM2, LDHA, and MCTs, that orchestrate glucose transport, glycolysis, and lactate production. As hub controllers of the glucose metabolic network, their aberrant overexpression directly drives tumor cell dependence on hyperactive glycolysis (Warburg effect), elevates lactate secretion, and contributes to extracellular acidification. These regulators are key components in mediating glucose metabolic reprogramming and the biosynthetic supply for ccRCC. Crucially, these specifically up-regulated metabolic proteins represent promising therapeutic targets due to their essential roles in ccRCC tumorigenesis and progression. Furthermore, their differential expression in cancerous versus normal tissues establishs a solid theoretical foundation for developing precision-targeted therapies against ccRCC (Fig. 2).

**Figure 3:**
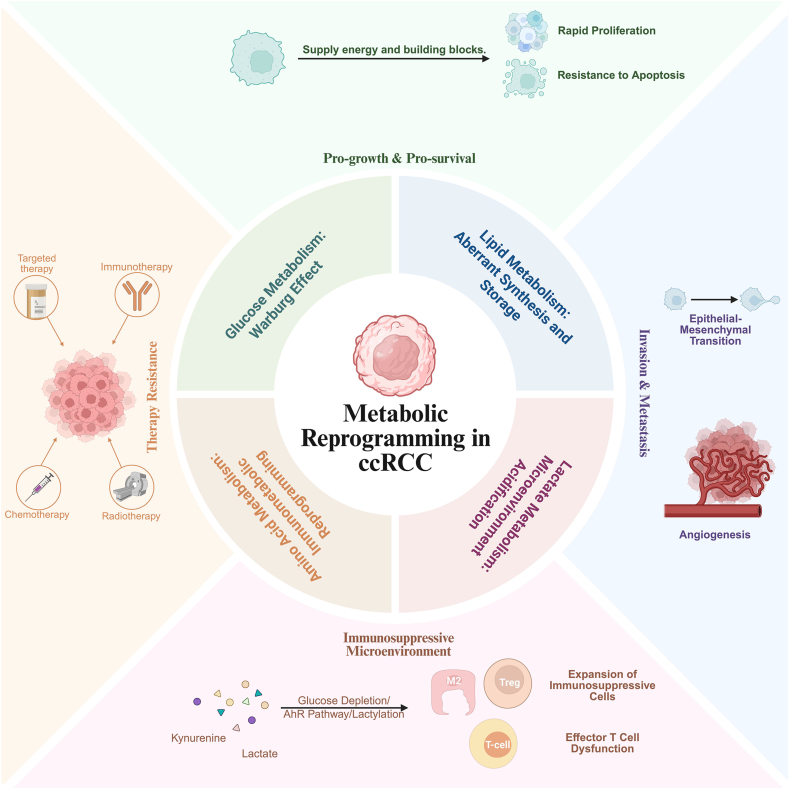
The multifaceted roles of metabolic reprogramming in ccRCC: From growth to immune evasion. This schematic diagram illustrates how VHL-deficient ccRCC cells rewire their core metabolic pathways—glucose, lipid, amino acid, and lactate metabolism—to fuel tumor progression. The reprogramming not only supports rapid proliferation by generating energy and biosynthetic precursors but also actively shapes an immunosuppressive tumor microenvironment. Key mechanisms include nutrient competition, release of oncometabolites (e.g., lactate, kynurenine), and induction of epigenetic modifications, which collectively drive T cell dysfunction, expansion of immunosuppressive cells (e.g., Tregs, M2 macrophages), and confer resistance to targeted therapy, immunotherapy, and chemotherapy.

## Membrane glucose transporter proteins

Glucose transporters, which belong to the solute carrier (SLC) superfamily, primarily comprise two class members: glucose transporters (GLUTs) and sodium-dependent glucose cotransporters (SGLTs). These membrane-embedded proteins mediate transmembrane glucose flux, with GLUTs facilitating gradient-dependent passive diffusion and SGLTs utilizing sodium electrochemical gradients for active transport.^14^ As the first critical step in glucose utilization, glucose transport is amplified in tumor cells due to the Warburg effect, which drives preferential glycolytic metabolism even under normoxia. This metabolic reprogramming coincides with up-regulated expression of all glycolytic enzymes.^13^ Compared to oxidative phosphorylation in normal cells, glycolysis generates substantially less ATP per glucose molecule. Paradoxically, proliferating tumor cells exhibit increased ATP demands. Along with this energy deficit, HIF-mediated inhibition of PDC also restricts glucose entry into the TCA cycle.^13^ Consequently, ccRCC cells compensate for their energy demands by dramatically enhancing glucose uptake via transporter up-regulation,^14^^,^^15^ creating a self-sustaining metabolic loop.

### GLUTs

GLUTs are a class of membrane proteins responsible for the initial step of glucose utilization in tumor cells. In renal carcinoma cells, persistent HIF-1α accumulation drives significantly elevated GLUT1 expression compared to normal renal tissues,^16^ thereby accelerating tumoral glucose uptake. Increased GLUT1 expression promotes tumor progression, while glucose deprivation or pharmacological inhibition of GLUT1 suppresses ccRCC proliferation and induces apoptosis.^17^ GLUT1 levels in ccRCC tumors are positively correlated with Fuhrman nuclear grade and poorer prognosis.^18^ Given its multifaceted roles in ccRCC pathogenesis, targeting GLUT1 represents a promising therapeutic strategy.

### SGLTs

SGLTs comprise two primary isoforms in humans: SGLT1 and SGLT2. SGLT1, predominantly expressed in intestinal epithelial cells, mediates glucose absorption from the gut lumen into the bloodstream. In contrast, SGLT2, localized to renal proximal tubular epithelia, facilitates glucose reabsorption from the glomerular filtrate, minimizing urinary glucose loss. Beyond its physiological roles, SGLT2 expression is up-regulated in multiple malignancies, including ccRCC, prostate, lung, and pancreatic cancers.^19^ Analogous to GLUTs, tumor cells leverage overexpressed SGLTs to augment glucose uptake, with SGLT2 inhibition suppressing proliferation and inducing apoptosis in cancer cells.^19^ Emerging evidence links elevated SGLT2 in renal tumors to adverse prognosis, while epidemiological studies have demonstrated that SGLT2 inhibitor use in diabetic patients is correlated with reduced renal cancer risk (adjusted HR 0.68; 95% CI, 0.58–0.81).^20^ Despite limited current research, intensified investigation of SGLT2 may provide critical insights into renal cancer metabolism and potential therapeutic target.

## Metabolic enzymes in aerobic glycolysis pathways

Following transport into cells via glucose transporters, glucose undergoes sequential enzymatic processing to generate ATP for cellular energy. In normal cells, intracellular glucose enters mitochondria where it fuels ATP production through the TCA cycle. In contrast, ccRCC cells, due to the reprogramming of glycolytic enzymes, drive glucose away from mitochondrial oxidation, instead of generating ATP cytoplasmically via the glycolytic pathway while converting pyruvate to lactate.

### Hexokinase

Hexokinase (HK), the initial rate-limiting enzyme in glucose metabolism, catalyzes the conversion of glucose to glucose-6-phosphate. While HK1 is ubiquitously expressed in most adult mammalian tissues, HK2 exhibits restricted high-level expression primarily in adipose, skeletal, and cardiac tissues.^21^ Tumor cells consistently exhibit increased HK2 expression, with ccRCC demonstrating particularly pronounced HK2 overexpression compared to normal renal tissue, driven by excessive HIF-1α-mediated transcriptional up-regulation.^22^ Elevated HK levels are positively correlated with chemoresistance and metastatic potential, while *in vitro* induction of HK2 expression enhances tumor invasiveness and proliferation,^16^ establishing HK2 as a promising therapeutic target in ccRCC.

### Phosphofructokinase

Phosphofructokinase (PFK), the second rate-limiting enzyme in the glycolytic pathway, catalyzes the conversion of fructose-6-phosphate (F-6-P) to fructose-1,6-bisphosphate (F-1,6-BP). PFK exists in multiple isoforms, among which PFK-1 is the core enzyme catalyzing this step. The activity of PFK-1 is dynamically regulated by metabolites, with fructose-2,6-bisphosphate (F-2,6-BP) being its most potent allosteric activator.^23^ The synthesis of F-2,6-BP is catalyzed by a distinct bifunctional enzyme called 6-phosphofructo-2-kinase/fructose-2,6-bisphosphatase (PFKFB). This enzyme generates F-2,6-BP by phosphorylating fructose-6-phosphate, thereby regulating PFK-1 activity and controlling overall glycolytic flux.^23^ Within the PFKFB family, PFKFB3 is the most prevalent isoform. It is overexpressed in various cancers, including ccRCC, and its elevated expression demonstrates significant associations with advanced-stage TNM classification and poor prognosis.^24^ Studies have demonstrated that knockdown of PFKFB3 impairs the glycolytic capacity in ccRCC cells, arrests cell cycle progression at the G1/S transition, and significantly delays tumor proliferation.^25^ Moreover, PFKFB4 is frequently overexpressed in ccRCC. Its knockdown not only suppresses tumor proliferation and invasion but also reverses resistance to Sunitinib.^26^ Consequently, targeting the PFKFB family, particularly PFKFB3 and PFKFB4, has emerged as a prominent strategy for metabolic intervention in ccRCC.

### Pyruvate kinase

Pyruvate kinase (PK), the terminal key enzyme in glycolysis, catalyzes the substrate-level phosphorylation converting phosphoenolpyruvate (PEP) to pyruvate and ATP. Mammals express four PK isoforms (PKL, PKR, PKM1, and PKM2), with PKM2 predominating in rapidly proliferating cells, including embryonic tissues, stem cells, and malignancies.^27^ PKM2 exists in dimeric and tetrameric conformations: tetramers dominate in normal cells, whereas tumor cells preferentially express the dimeric form.^27^ The low-activity dimeric PKM2 restricts PEP-to-pyruvate conversion, shunting glycolytic intermediates into branching pathways that fuel tumor biomass synthesis.^28^ Crucially, induction of tetramer formation reverses aerobic glycolysis, depletes biosynthetic precursors, and suppresses tumor growth.^27^ Thus, PKM2 overexpression not only diverts glycolytic intermediates for enhanced biosynthetic processes, but also activates mTOR signaling, which drives the anabolic utilization of these metabolites to fuel tumor growth.^29^ Given PKM2 overexpression in ccRCC and its kinase-dependent activation of the PI3K/AKT/mTOR axis, targeting PKM2 represents a promising strategy to disrupt anabolic metabolism and halt disease progression.

### Pyruvate dehydrogenase complex

PDC is a multi-enzyme assembly that catalyzes the conversion of pyruvate to acetyl-CoA. Comprising three core enzymes, pyruvate dehydrogenase (E1), dihydrolipoyl transacetylase (E2), and dihydrolipoyl dehydrogenase (E3), along with six essential cofactors (thiamine pyrophosphate, lipoic acid, FAD, NAD+, CoA, and Mg2+), this complex gate carbon entry into the TCA cycle. Crucially, pyruvate dehydrogenase (PDH) serves as the rate-limiting component, generating acetyl-CoA for oxidative metabolism. The activity of PDH is dynamically regulated by opposing kinases and phosphatases: pyruvate dehydrogenase kinases (PDKs) inactivate PDH through phosphorylation, while pyruvate dehydrogenase phosphatases (PDPs) restore activity via dephosphorylation. Among PDK isoforms (PDK1-4), HIF-1α directly transactivates the PDK1 gene, driving its pronounced overexpression.^30^ Elevated PDK1 inactivates PDH, diverting pyruvate away from the TCA cycle toward lactate production. In ccRCC, constitutive HIF-1α stabilization due to VHL inactivation induces significant PDK1 up-regulation compared to adjacent normal tissue.^31^ This metabolic rewiring establishes PDK inhibition as a promising therapeutic strategy for ccRCC.

## Key enzymes in regulation of lactate metabolic reprogramming in ccRCC

Concomitant with active glycolysis, ccRCC cells achieve suboptimal ATP yield to fuel their growth, infiltration, and migration while generating substantial intracellular lactate accumulation. This lactate surplus profoundly disrupts intracellular homeostasis, prompting tumor cells to actively export excess lactate to maintain acid–base equilibrium. The dual processes of hyperproduction and efflux of lactate constitute a defining hallmark of metabolic reprogramming in ccRCC (Fig. 2).

## Lactate dehydrogenase

Lactate dehydrogenase (LDH), catalyzing the interconversion of pyruvate and lactate through redox reactions, comprises five isoenzymes in humans that are regulated by HIF-1 and c-Myc.^32^ Concomitant with diminished glucose entry into the TCA cycle and hyperactive glycolysis, pyruvate accumulation occurs, while HIF-1α-driven LDHA up-regulation promotes pyruvate-to-lactate conversion.^32^ ccRCC tissues exhibit significantly elevated LDHA expression compared with normal kidney tissues, with increased levels correlating inversely with both disease-free survival (DFS) and overall survival (OS).^33^^,^^34^ Enhanced LDHA expression potentiates tumor cell growth and migratory capacity, whereas its down-regulation suppresses proliferation, impairing tumor invasive potential and concurrently disrupting immune evasion.^34^ Thus, targeting LDHA to inhibit pyruvate–lactate flux represents an emerging therapeutic approach in ccRCC.

## Monocarboxylate transporters

Intracellular lactate accumulation in tumor cells coincides with upregulated expression of lactate transporter genes (*MCT1* and *MCT4*), enabling massive lactate efflux.^35^ This extracellular lactate pool impairs CD8+ T-cell proliferation and activation, facilitating tumor immune evasion.^36^ Concurrently, the acidified tumor microenvironment stimulates vascular endothelial growth factor (VEGF) secretion and angiogenesis,^36^ accelerating oncogenesis. Elevated MCT expression in renal cancer patients is significantly correlated with poorer prognosis and shortor OS.^37^ Enhanced expression of MCT1 and MCT4 potentiates proliferative and invasive capacities in ccRCC. Pharmacological blockade of MCTs reduces extracellular lactate levels, attenuating tumor cell proliferation and invasion,^38^ while suppressing intratumoral angiogenesis.^35^

## Key regulators of lipid metabolic reprogramming in ccRCC

One of the most remarkable metabolic features of ccRCC is its profound lipid metabolic reprogramming. This is centrally characterized by the abnormal and massive accumulation of lipids, particularly triglycerides and cholesteryl esters, within tumor cells. This accumulation is not merely passive storage; rather, it results from cancer cells actively remodeling its metabolic network to support their rapid proliferation. This reprogramming involves significant alterations in the expression and function of key enzymes involved in fatty acid and ketone body metabolism, which collectively drive tumor progression. In exploring novel therapeutic strategies for ccRCC, the role of lipid metabolic reprogramming has gained increasing prominence.

## Metabolic enzymes in fatty acid metabolism

ccRCC cells consistently exhibit a heightened dependence on fatty acids, revealing potential therapeutic vulnerabilities. Lipid droplet biogenesis is pivotal in ccRCC pathogenesis, histologically manifesting as the characteristic eosinophilic clear cytoplasm. The fatty acids stored in lipid droplets help to maintain the integrity of the endoplasmic reticulum, prevent lipid peroxidation, and enhance the metastatic potential of tumors.^39^^,^^40^ Notably, multiple fatty acid metabolic enzymes demonstrate up-regulated expression in ccRCC, which is significantly correlated with adverse clinical outcomes.^41^ This mechanistic evidence establishes fatty acid metabolism as a promising therapeutic frontier for ccRCC (Fig. 2).

### ATP-citrate lyase

ATP-citrate lyase (ACLY) functions as a critical metabolic nexus linking glucose and lipid metabolism by converting citrate, a TCA cycle intermediate, into acetyl-CoA to fuel *de novo* lipogenesis.^42^ In ccRCC, ACLY expression is significantly elevated compared to adjacent normal tissues and is positively correlated with advanced T-stage and higher Fuhrman nuclear grade. Mechanistically, ACLY overexpression remodels the tumor immune microenvironment through impaired immune cell infiltration, thereby facilitating immune evasion.^43^ Conversely, ACLY knockdown disrupts lipid metabolic reprogramming, simultaneously suppressing cancer cell proliferation and inducing apoptosis.^44^ These converging lines of evidence nominate ACLY as a promising therapeutic target in ccRCC.

### Acetyl-CoA carboxylase

Acetyl-CoA carboxylase (ACC) functions as a critical metabolic regulator through its two isoforms with compartmentalized and antagonistic roles: cytosolic ACC1 (ACCA) serves as the rate-limiting enzyme in *de novo* lipogenesis, catalyzing the conversion of ACLY-derived acetyl-CoA to malonyl-CoA to fuel tumor growth and proliferation^44^; whereas mitochondrial ACC2 (ACCB) generates malonyl-CoA that inhibits carnitine palmitoyltransferase 1 (CPT1), thereby gating fatty acid β-oxidation flux.^45^ Transcriptomic analyses demonstrate elevated ACCA mRNA levels in ccRCC tumors relative to non-malignant renal tissues, with ACC overexpression correlating significantly with reduced OS (adjusted HR = 1.73, 95% CI 1.25–2.41).^46^ Crucially, AMPK-mediated phosphorylation at Ser79 inhibits ACC activity, suppressing fatty acid synthesis during energy depletion.^47^

### Fatty acid synthase

Fatty acid synthase (FASN), the pivotal enzyme in *de novo* lipogenesis, catalyzes the condensation of acetyl-CoA and malonyl-CoA into palmitoyl-CoA to govern long-chain fatty acid elongation.^48^ In ccRCC, FASN protein expression is significantly up-regulated and strongly correlated with aggressive clinicopathological features—including high Fuhrman grade, advanced clinical stage, larger tumor size, propensity for metastasis, and reduced cancer-specific survival (CSS)—collectively portending poor prognosis.^41^^,^^49^ The oncogenic role of FASN, validated across diverse malignancies,^39^ extends to ccRCC where pharmacological inhibition robustly suppresses tumor cell proliferation,^44^^,^^50^ establishing FASN as a key therapeutic vulnerability.

### Stearoyl-CoA desaturase 1

Stearoyl-CoA desaturase (SCD), the rate-limiting enzyme converting saturated fatty acids (SFAs) to monounsaturated fatty acids (MUFAs), exists as SCD1 and SCD5 isoforms in humans. While SCD5 exhibits restricted tissue distribution and remains poorly characterized, SCD1, the predominant isoform, is widely expressed and performs pro-tumorigenic functions across multiple cancers.^51^ In ccRCC, high SCD1 overexpression is significantly correlated with adverse clinicopathological parameters, including OS (HR 2.94, 95% CI 1.44–6.03, *P* = 0.003), advanced TNM stage (*P* = 0.021), positive nodal status (pN stage, *P* = 0.014), higher Fuhrman grade (P = 0.014), and larger tumor size (*P* = 0.040).^52^ As a key metabolic regulator, SCD1 catalyzes MUFA synthesis to maintain SFA/MUFA homeostasis—a balance critical for oncogenesis. Rapidly proliferating tumor cells require MUFAs as essential precursors for phospholipid, triacylglycerol, and cholesteryl ester biosynthesis, explaining the elevated MUFA-rich lipid content and concomitant reduction in SFA/PUFA levels observed in diverse tumors.^53^ Moreover, MUFAs resist peroxidation due to their lack of bis-allylic moieties, conferring ferroptosis resistance.^54^ Notably, SCD1 is universally overexpressed in ccRCC tumors versus adjacent normal tissues. Functional studies have demonstrated that SCD1 knockdown or pharmacological inhibition induces apoptosis in ccRCC models,^55^ establishing SCD1 as a compelling therapeutic target.

### CD36

CD36, a transmembrane glycoprotein, facilitates the uptake of hydrophobic molecules, including fatty acids, phospholipids, and cholesterol, orchestrating lipid trafficking via intracellular and extracellular vesicles to regulate fatty acid metabolism and energy homeostasis.^56^ This scavenger receptor is frequently up-regulated across malignancies, critically promoting tumor proliferation and metastasis.^56^ CD36 is significantly overexpressed in ccRCC,^57^ where it potently enhances intracellular lipid accumulation while accelerating cancer cell proliferation and migration.^58^ Mechanistically, HIF-2α directly transactivates CD36 under hypoxic conditions, a response abrogated by the HIF-2α inhibitor PT2385,^58^ establishing CD36 as a hypoxia-inducible effector linked to VHL inactivation and revealing its therapeutic potential in ccRCC.

### CPT1A

Mitochondrial fatty acid β-oxidation represents the primary catabolic pathway for fatty acids, with CPT1 on the outer mitochondrial membrane serving as its critical gatekeeper. Among the three CPT1 isoforms (CPT1A, CPT1B, and CPT1C), reduced CPT1A expression in ccRCC patients is associated with poor OS, larger tumor size, and higher tumor grade.^59^ Mechanistically, the hypoxia-inducible factors HIF-1α and HIF-2α directly suppress CPT1A through promoter hypermethylation, reducing fatty acid oxidation and promoting lipid accumulation—effects that are reversible upon exogenous CPT1A restoration.^60^ Pharmacological activation of CPT1A attenuates lipid deposition while suppressing ccRCC cell proliferation and migration.^61^ Moreover, CPT1A activation not only enhances fatty acid breakdown but also inhibits CD36-mediated fatty acid uptake, thereby further reducing intracellular lipid accumulation.^62^ Collectively, CPT1A functions as both a prognostic biomarker and promising therapeutic target in ccRCC.

## Metabolic enzymes in ketone body metabolism

Beyond the well-established dependencies on *de novo* lipogenesis and fatty acid uptake, the rewiring of lipid metabolism in ccRCC extends to its catabolic processes, particularly ketone body metabolism. However, current research on the role of ketone body metabolism in ccRCC remains in its preliminary stages, and the specific regulatory mechanisms governing its synthesis and utilization are not yet fully understood. In many types of tumors, ketone body metabolism exhibits a unique pattern characterized by “emphasizing utilization over generation”. This is manifested as a decrease in the expression of enzymes involved in ketone body synthesis (such as HMGCS2, HMGCL, and ACAT1) and an increase in the expression of enzymes responsible for ketone body breakdown (such as OXCT1 and BDH1)^63^, ^64^, ^65^ (Fig. 2). *In vitro* experiments have demonstrated that supplementation with β-hydroxybutyrate (BHB) can significantly inhibit the proliferation of ccRCC cells.^66^ Furthermore, *in vivo* studies have revealed that a ketogenic diet not only suppresses the growth of ccRCC xenografts in mouse models but also exhibits a synergistic enhancement effect when combined with immunotherapy.^67^ The evidence suggests that the intracellular accumulation of ketone bodies may exert inhibitory effects on ccRCC cells, thereby impeding tumor progression. ccRCC exhibits significant reprogramming of ketone body metabolism, primarily characterized by the down-regulation or functional inactivation of ketogenic enzymes, which is closely associated with the malignant behavior of the tumors. Specifically, HMGCS2, a key rate-limiting enzyme in ketone body synthesis, shows significantly reduced expression in ccRCC.^68^ This low expression state is correlated with poor patient prognosis, and studies have found that its down-regulation is associated with promoter hypermethylation.^68^ Functionally, restoring HMGCS2 expression effectively inhibits the migration and invasion capabilities of ccRCC cells and reverses the epithelial–mesenchymal transition (EMT) process.^68^ Similarly, the inactivation of HMGCL is also associated with unfavorable patient prognosis.^69^ Another key ketone body synthesis enzyme, ACAT1, also shows down-regulated expression in ccRCC.^68^^,^^70^ Clinical sample analysis further indicates that the low expression level of ACAT1 in ccRCC tissues is closely associated with disease progression. Functional experiments have confirmed that overexpressing ACAT1 can significantly inhibit the migration, invasion, and proliferation of ccRCC cells *in vivo* and induce tumor cell apoptosis.^70^ Based on the lipid metabolic features of ccRCC, it is plausible to speculate that there may be an up-regulation of ketolytic enzyme expression; however, this hypothesis awaits further validation in future studies.

## Key regulators of amino acid metabolic reprogramming in ccRCC

In ccRCC, the reprogramming of amino acid metabolism represents a core feature underpinning malignant progression, with particularly notable abnormalities in the metabolism of glutamine, tryptophan, and arginine. These metabolic alterations, such as VHL deletion and HIF signaling pathway activation, are closely associated with key molecular events in ccRCC. Through changes in the expression of key metabolic enzymes, they collectively promote tumor proliferation, adaptation to the microenvironment, and immune evasion. The following sections systematically elaborate on the metabolic remodeling of these three amino acids in ccRCC and the roles of their key regulatory factors.

## Metabolic enzymes in glutamine metabolism

Glutamine, while categorized as a non-essential amino acid, is indispensable for neoplastic proliferation, with tumor cells predominantly channeling glucose into glycolytic lactate export via anaerobic oxidation. This amino acid undergoes sequential enzymatic conversion: initially hydrolyzed to glutamate by glutaminase (GLS), and then transformed to α-ketoglutarate (α-KG) through either oxidative deamination via glutamate dehydrogenase (GLUD1) or transamination, ultimately fueling the TCA cycle.^71^ As a fundamental anabolic precursor in ccRCC, exogenous glutamine deprivation substantially depletes intracellular lipid droplets and suppresses proliferation—effects markedly attenuated under glucose restriction.^72^ Crucially, VHL-mutant ccRCC xenograft models have demonstrated that pharmacological inhibition of GLS or transaminases attenuates glutaminolysis and curtails tumor growth,^73^ collectively establishing glutamine metabolism as a compelling therapeutic frontier in RCC management (Fig. 2).

### Solute carrier family 1, member 5

SLC1A5 is significantly overexpressed in ccRCC, primarily driven by HIF-2α activation following VHL inactivation.^74^ This elevated expression is strongly correlated with advanced TNM stage (*P* = 0.032) and higher Fuhrman nuclear grade (*P* = 0.015).^75^ Clinically, increased SLC1A5 independently associates with worse OS (HR > 1, *P* < 0.001), though no significant difference was observed in recurrence-free survival (RFS, *P* = 0.083).^75^ Critically, functional inhibition of SLC1A5 suppresses proliferation while impairing invasion and migration capabilities in human ccRCC cell lines (A498 and Caki1).^76^ Consequently, SLC1A5 has emerged as a HIF-2α-driven metabolic vulnerability that integrates molecular pathogenesis, clinical aggressiveness, and actionable therapeutic targeting in ccRCC.

### Glutaminase 1

GLS is a mitochondrial enzyme whose primary function is to catalyze the conversion of glutamine to glutamate, encompassing two isoforms: GLS1 and GLS2. In most tumors, GLS1 expression is up-regulated, while GLS2 expression is down-regulated.^77^ GLS1 has two splicing variants, GAC and KGA, with GAC exhibiting higher enzymatic activity in cancers.^78^ Compared to normal kidney tissue, RCC cells express higher levels of GLS1, particularly the splicing variant GAC, resulting in therapeutic sensitivity to GLS inhibition.^79^ Multiple GLS1 inhibitors have been identified to effectively block tumor cells' utilization of glutamine, demonstrating antitumor efficacy in both *in vitro* and *in vivo* studies.^73^^,^^79^ Consequently, targeted inhibition of GLS1 has emerged as a promising therapeutic strategy for metabolic intervention in ccRCC.

## Metabolic enzymes in tryptophan metabolism

As an essential amino acid, the dysregulation of tryptophan metabolism represents a key mechanism of tumor immune evasion. In various malignancies, including RCC, tryptophan is excessively consumed, while the levels of its metabolite, kynurenine, are significantly elevated.^80^ This phenomenon is primarily attributed to the aberrant overexpression of the rate-limiting enzymes indoleamine 2,3-dioxygenase (IDO) and tryptophan 2,3-dioxygenase (TDO)^81^^,^^82^ (Fig. 2). Tryptophan depletion directly suppresses T-cell function. Concurrently, the accumulated bioactive metabolites such as kynurenine, activate signaling pathways like the aryl hydrocarbon receptor (AhR), thereby cooperatively shaping an immunosuppressive microenvironment and driving malignant tumor progression.^83^^,^^84^ Consequently, targeting IDO and TDO has emerged as a highly promising anticancer strategy.

### Indoleamine 2,3-dioxygenase

IDO is a cytosolic heme enzyme that serves as the key rate-limiting enzyme in the tryptophan metabolic pathway, responsible for catalyzing the conversion of tryptophan to kynurenine. This enzyme comprises two subtypes, IDO1 and IDO2, with current research primarily focused on IDO1. In ccRCC, the expression of IDO1 is significantly up-regulated and drives immune escape by initiating dual immunosuppressive mechanisms: first, it catalyzes tryptophan depletion to activate the GCN2 pathway, resulting in the inactivation of effector T cells; second, it generates kynurenine to activate the AhR pathway, promoting the differentiation and infiltration of regulatory T cells (Tregs). These two mechanisms act synergistically to collectively establish an immunosuppressive microenvironment in ccRCC.^85^^,^^86^ Following treatment with immune checkpoint inhibitors (ICBs), an up-regulation of IDO1 expression has been observed in ccRCC tissues, suggesting its potential role in mediating resistance to immunotherapy.^86^^,^^87^

### Tryptophan 2,3-dioxygenase

TDO functionally resembles IDO1, serving as a key enzyme that catalyzes the conversion of tryptophan into kynurenine. In advanced RCC, TDO expression is significantly elevated and is associated with poor patient prognosis and resistance to immunotherapy.^88^^,^^89^ Similar to IDO1, TDO operates through an identical mechanism: it mediates tryptophan depletion and kynurenine accumulation, thereby activating the AhR pathway.^88^^,^^90^ This leads to the functional impairment of effector T cells and promotes the differentiation and infiltration of Tregs, collectively shaping an immunosuppressive microenvironment in ccRCC. Notably, a complementary relationship exists between TDO and IDO1. Studies indicate that inhibiting or knocking down IDO1 can trigger an up-regulation of TDO expression as a compensatory mechanism, whereas simultaneously inhibiting both IDO1 and TDO can synergistically enhance antitumor immune effects.^91^, ^92^, ^93^ This functional redundancy and metabolic compensation likely constitute a major reason for the failure of IDO1 inhibitor monotherapy to achieve the expected efficacy in clinical trials.

## Metabolic enzymes in arginine metabolism

Arginine is an important semi-essential amino acid. Under normal physiological conditions, the body can meet its own demands through the urea cycle without requiring additional supplementation. However, under certain physiological or pathological conditions, such as during periods of growth or disease, the demand for arginine increases significantly. When endogenous synthesis cannot meet this heightened demand, the body becomes reliant on exogenous sources for arginine supply.^94^ In cancer therapy, arginine plays a complex “double-edged sword” role. The core contradiction lies in the following: On the one hand, many tumor cells, due to deficiencies in the urea cycle enzymes ASS1 and ASL, become “arginine auxotrophic” (Fig. 2). This means that they depend on exogenous arginine to sustain their proliferation and energy metabolism. This dependency makes them vulnerable to arginine deprivation therapy (e.g., using agents like ADI-PEG 20), which aims to selectively “starve” the tumor cells by depleting systemic arginine. On the other hand, arginine is also an essential nutrient for the activation, proliferation, and cytotoxic function of T cells. Its depletion can directly lead to T-cell dysfunction and cell cycle arrest, thereby potentially hampering anti-tumor immunity.^95^ In ccRCC, there is a prevalent reduction or loss of ASS1 and ASL expression. This deficiency in the urea cycle prompts the tumor cells to redirect aspartate towards pyrimidine synthesis, thereby supporting rapid proliferation.^96^ Experimental evidence shows that restoring the expression of ASS1 and ASL can effectively inhibit the growth of ccRCC *in vivo*.^96^ Consequently, depleting arginine has emerged as a potentially beneficial therapeutic strategy for ccRCC, primarily targeting the metabolic vulnerability of the ASS1/ASL-deficient tumor cells.

## Targeting glucose metabolic reprogramming in ccRCC

Advances in deciphering the molecular underpinnings of glucose metabolic reprogramming in ccRCC have established its core hallmark—preferential reliance on glycolysis for energy production even under oxygen-replete conditions (Warburg effect) as a key driver of malignant progression. This metabolic rewiring is predominantly orchestrated by aberrant activation of the VHL-HIF signaling axis and involves dysregulation of critical enzymes (e.g., HK2, PKM2, LDHA) and transporters (e.g., GLUT1, MCT1/4). These effectors not only reconfigure cellular bioenergetics and anabolic processes but also directly potentiate proliferation, invasion, and immune evasion. Consequently, targeting these nodal points in glucose reprogramming represents a highly promising therapeutic frontier for ccRCC. Currently, innovative agents against these targets are actively advancing through rigorous investigation, demonstrating significant potential to modulate the tumor metabolic microenvironment and suppress oncogenesis. The following delineates key progress in the drug development targeting ccRCC glucose metabolism.

## Targeting glucose transport

The Warburg effect renders ccRCC cells profoundly inefficient in energy production compared to normal tissues. As a metabolically hyperactive and proliferative malignancy, ccRCC exhibits extraordinary glucose demands. To fuel its accelerated growth and heightened metabolic activity, ccRCC cells dramatically up-regulate glucose uptake. This insatiable glucose requirement drives dysregulated expression of glucose transporters (GLUTs and SGLTs), establishing their overexpression as a distinguishing pathological feature in ccRCC versus normal cells.^127^ Consequently, pharmacological blockade of these transporters represents a promising therapeutic strategy to constrain tumor progression (Table 1).

**Table 1:** Potential pharmacological targets and inhibitors targeting glucose metabolism.

Drug target	Mechanism of action	Small-molecule targeting agents	Research phase	Reference
GLUT1	Transport glucose across membrane	WZB117 DRB18 STF-31 Fasentin Glutor KL-11743 NV-5440	Preclinical Preclinical Preclinical Preclinical Preclinical Preclinical Preclinical	^97^ ^98^ ^99^ ^100^ ^101^ ^102^ ^103^
SGLT2	Transport glucose across membrane	Dapagliflozin Canagliflozin	Preclinical Preclinical	^104^^,^^105^ ^106^
HK2	Convert glucose to glucose-6-phosphate	2-DG WP1112 3-BrPa LND BNBZ Sinominine IKA	Preclinical Phase I Preclinical Preclinical Preclinical Preclinical Preclinical	^107^ ^108^^,^^109^ ^110^ ^111^ ^112^ ^113^ ^114^
PFKFB3	Phosphorylate fructose-6-phosphate to fructose-2,6-bisphosphate	3-PO PFK-15 PFK-158 KAN0438757	Preclinical Preclinical Phase I Preclinical	^115^ ^116^ ^117^ ^118^
PKM2	Converts PEP to pyruvate with concomitant ATP production	TLN-232 Shikonin BEN PTL	Phase II Preclinical Preclinical Preclinical	^119^ ^120^^,^^121^ ^122^ ^123^
PDK1	Inhibit the activity of PDC by phosphorylation	DCA Hordenine JX06	Phase II Preclinical Preclinical	^124^ ^125^ ^126^

Comment: Of the inhibitors listed in this table, the most promising in terms of translational potential include: (1) SGLT2 inhibitors (such as Dapagliflozin and Canagliflozin), which are already approved glucose-lowering agents with well-established safety profiles, making them highly amenable to repurposing for ccRCC therapy. Preclinical studies have demonstrated their ability to induce apoptosis in tumor cells; (2) PFK-158, an inhibitor of PFKFB3, has entered Phase I clinical trials (NCT02044861). Although its development was halted due to insufficient efficacy, it provides valuable insights for combination therapy approaches; (3) Novel inhibitors such as BNBZ (an HK2 inhibitor), which have shown high potency and low toxicity in preclinical models. Overall, the selection of these inhibitors is based on their specific targeting of key metabolic nodes in ccRCC, with SGLT2 inhibitors being prioritized for further investigation due to their clinical availability.

## Targeting GLUTs

Multiple small molecules selectively inhibit GLUTs, encompassing natural extracts (alkaloids, flavonoids), heterocyclic compounds, and phenolics.^128^ Among the 25 natural compounds demonstrating anticancer activity in tumor models include phloretin,^129^ genistein,^130^ curcumin,^131^ Polyphenol,^132^ quercetin,^133^ etc. Polyphenols can suppress glucose uptake and proliferation in cancer cells.^132^ Genistein induces renal cancer cell apoptosis by up-regulating CDKN2a expression and reducing its methylation,^134^ while also inhibiting tumor growth/metastasis via miR-1260b down-regulation and Wnt signaling suppression.^135^ Synthetic GLUT inhibitors exhibit anticancer effects in preclinical models by blocking glucose uptake. WZB117, a polyphenol-derived inhibitor, reduces GLUT1 expression and glycolytic activity, impairing the tumor energy supply, though its aqueous instability limits its clinical application.^97^ Comparatively, the novel inhibitor DRB18 demonstrates superior stability and enhanced glucose uptake inhibition across tumor cell lines.^98^ STF-31 selectively targets GLUT1, suppressing glucose uptake and growth in ccRCC and other glycolytic tumors.^99^ Normal renal cells exhibit low toxicity sensitivity due to non-reliance on GLUT1-mediated transport, yet ubiquitous GLUT1 expression in healthy tissues (e.g., erythrocytes) constrains their clinical utility.^14^ Fasentin inhibits glucose uptake by binding GLUT1/GLUT4. Recent studies have revealed its anti-angiogenic potential through endothelial proliferation suppression,^100^ though its safety requires validation. Glutor—a novel pan-GLUT inhibitor targeting GLUT1/2/3—shows potent antitumor effects across models with favorable safety profiles.^101^ KL-11743 specifically blocks cancer cell glucose uptake *in vitro*, with enhanced efficacy in TCA cycle-deficient cell lines.^102^ NV-5440 dually inhibits mTORC1 and GLUT1, effectively suppressing glucose uptake and tumor proliferation. Its *in vivo* stability suggests therapeutic potential for dual-targeting metabolic intervention in ccRCC.^103^

To date, no GLUT inhibitors have been evaluated in clinical trials for RCC. GLUT inhibitors currently still face some issues, such as the widespread distribution of GLUT in the body, which can easily affect normal cells upon application. Moreover, due to the heterogeneity of tumor cells, the varying expression levels of different GLUT subtypes may impact the antitumor efficacy of GLUT inhibitors.^14^ The clinical application of GLUT inhibitors is currently still subject to significant limitations and requires further exploration.

## Targeting SGLTs

SGLT-2 inhibitors are currently approved primarily for glycemic control in diabetic patients. Research has revealed that these drugs exhibit inhibitory effects on various tumors, including liver cancer, pancreatic cancer, and prostate cancer.^136^ *In vitro* studies have demonstrated that dapagliflozin, as an SGLT-2 inhibitor, can reduce the viability of renal cancer cells, promote tumor cell apoptosis, and diminish tumor volume.^104^^,^^105^ Additionally, studies indicate that canagliflozin promotes AMPK activity, inhibits the MAPK and mTOR-p70S6k/4EBP1 pathways, activates cell cycle checkpoints, and suppresses tumor proliferation through partial inhibition of HIF-1α.^106^ The antitumor effects of SGLT-2 inhibitors have been validated across multiple tumor models, suggesting their potential as anticancer agents.^19^ As widely used antidiabetic drugs, SGLT-2 inhibitors possess well-established safety profiles, a significant advantage for their repurposing as potential antitumor therapeutics. Nevertheless, the precise mechanisms underlying their anticancer effects remain debated and warrant further investigation.

## Targeting rate-limiting enzymes of glycolysis

In renal cancer cells, glucose is primarily metabolized through the glycolytic pathway, a process abnormally active in tumor cells to meet their ever-increasing energy demands. To cope with this high energy consumption, tumor cells must enhance their glycolytic efficiency, during which the expression levels of key glycolytic enzymes become significantly up-regulated, a feature particularly striking compared to normal cells.^11^ Normal cells predominantly rely on the TCA cycle for energy production, a metabolic pathway far more efficient in generating energy. This metabolic divergence creates opportunities for targeted renal cancer therapy. By inhibiting key enzymes in glycolysis, the energy supply of tumor cells can be effectively blocked, thereby suppressing their proliferation. Given that normal cells depend more on the TCA cycle, such a therapeutic strategy targeting glycolytic enzymes has relatively minor impact on normal cells, which consequently reduces treatment-related side effects. Therefore, targeting key enzymes in the glycolytic pathway of renal carcinoma cells not only promises effective tumor suppression but also potentially enhances treatment safety while preserving normal cellular functions. This therapeutic approach holds broad application prospects for future research and clinical trials (Table 1).

## Targeting HK

The glucose analog 2-deoxy-d-glucose (2-DG) is catalyzed by HK2 upon cellular entry and is converted to 2-deoxy-d-glucose-6-phosphate (2-DG-6-P), thereby blocking glycolysis at its initial stage.^107^ Early studies demonstrated that 2-DG monotherapy yielded suboptimal outcomes in cancer treatment, yet it enhances therapeutic efficacy when combined with other agents.^107^ An *in vitro* study on 2-DG combined with tyrosine kinase inhibitors revealed that this combination reduces ATP production and increases the sensitivity of ccRCC to pazopanib treatment.^107^ WP1112, a novel 2-DG analog, demonstrates superior anticancer effects compared to 2-DG while exhibiting favorable tolerability in murine models. It has entered Phase I clinical trials for treating glioblastoma multiforme.^108^^,^^109^ 3-Bromopyruvate (3-BrPa), a highly reactive pyruvate analog, inhibits tumor glycolysis as an HK2 inhibitor, inducing apoptosis through ATP depletion.^110^ *In vitro* studies confirmed that primary ccRCC cells are sensitive to 3-BrPa, which inhibits glycolysis and induces apoptosis at concentrations tolerated by normal primary renal cells.^110^ Recent research has combined 3-BrPa with nanomaterials to enhance tumor sensitivity to radiotherapy and photodynamic therapy while reducing adverse effects.^137^^,^^138^ Lonidamine (LND), an indole derivative, inhibits glycolysis as an HK-2 inhibitor. Similar to 2-DG, its monotherapy shows limited efficacy. However, combination studies have revealed that LND sensitizes various tumors to chemo-radiotherapy.^111^ Current research integrates LND with liposomes (LPs) to form nanomedicine, leveraging nanoparticle accumulation in tumors to enhance efficacy and reduce toxicity.^139^, ^140^, ^141^ Benitrobenrazide (BNBZ), a novel HK inhibitor, directly binds HK2, significantly suppressing tumor glycolysis and proliferation.^112^ With high efficacy and low toxicity, BNBZ represents a promising antitumor agent.^112^ Additionally, several new HK2 inhibitors, including sinominine^113^ and Ikarugamycin (IKA),^114^ have demonstrated potent tumor suppression *in vitro*, suggesting their therapeutic potential for ccRCC.

## Targeting PFKFB

Researchers have identified PFKFB3 as a potential therapeutic target and developed inhibitors against this enzyme to disrupt tumor glucose metabolism for cancer treatment.^25^ These inhibitors suppress PFKFB3 activity, reduce fructose-2,6-bisphosphate levels, and consequently inhibit tumor growth and metastasis, exemplified by agents like 3-PO,^115^ PFK-15^116^ and PFK-158.^142^ As a first-generation PFKFB3 inhibitor, 3PO reduces metabolic flux by inhibiting key glycolytic enzymes. However, due to its low target selectivity and multi-pathway mechanisms of action, its antitumor and anti-inflammatory effects are partially dependent on non-PFKFB3 pathways. This complexity established 3PO as the foundation for optimizing subsequent high-selectivity inhibitors (e.g., PFK-15, PFK-158).^143^ Compared to the first-generation PFKFB3 inhibitor 3-PO, the derivative PFK-15 exhibits enhanced selectivity and potency, and demonstrates significantly stronger anti-tumor efficacy in multiple tumor models.^116^^,^^144^ As the latest-generation structural derivative of 3PO, PFK-158 demonstrates significantly higher biological potency against PFKFB3 than PFK-15 and exhibits lower systemic toxicity in *in vivo* models.^117^ It entered Phase I clinical trials for advanced solid malignancies (NCT02044861) in 2014, showing no serious adverse events during one-year follow-up, though the trial was discontinued due to insufficient efficacy.^117^ The novel PFKFB3 inhibitor KAN0438757, a phenylsulfonamide-based compound, significantly reduced the migration and invasion capabilities in colon cancer cells, inhibiting tumor aggressiveness without apparent toxicity to normal tissues or healthy mice.^118^ However, its therapeutic potential in renal cancer requires further investigation. Current PFKFB4 inhibitors remain scarce. Compound 5MPN exhibits antitumor activity by suppressing tumor PFKFB4.^145^ The ongoing development of novel PFKFB inhibitors may potentially offer additional therapeutic options for metastatic ccRCC patients.

## Targeting PKM2

TLN-232, a PKM2 inhibitor, is currently undergoing Phase II clinical trials (NCT00422786) for metastatic RCC. It targets PKM2 to terminate glycolysis, thereby suppressing tumor proliferation.^119^ Shikonin, another PKM2 inhibitor, has also demonstrated growth-suppressive effects on renal cancer cells i*n vitro*.^120^^,^^121^ Additionally, it enhances sunitinib sensitivity in drug-resistant RCC cells by inhibiting the AKT/mTOR pathway.^146^ While shikonin significantly inhibits RCC cell growth, its efficacy varies across different subtypes, warranting further investigation into its therapeutic potential.^146^ Benserazide (BEN), a novel PKM2 inhibitor, directly binds to and inactivates PKM2 in melanoma and colon cancer models. This suppresses glycolysis, promotes tumor cell apoptosis, and inhibits tumor growth.^122^ The investigational compound 3h potently inhibits both glycolysis and mitochondrial respiration in tumor cells, inducing dual apoptosis and autophagic cell death.^147^ Notably, activating the tetrameric form of PKM2 to restore its pyruvate kinase activity may reverse the Warburg effect and inhibit tumorigenesis.^148^ A series of parthenolide (PTL) derivatives exhibit PKM2-activating properties, with 29e demonstrating potent activity by promoting the dimer-to-tetramer transition of PKM2, significantly suppressing tumor growth *in vivo* and *in vitro*.^123^ Other activators, including TEPP-46, quinolone sulfonamide, and DASA-58, also enhance PKM2’s pyruvate kinase activity and inhibit growth in murine tumor models.^149^

## Targeting PDK

Dichloroacetate (DCA), an inhibitor of PDK1, has demonstrated in preclinical studies its ability to reduce HIF transcriptional activity, inhibit intratumoral angiogenesis, and reverse aerobic glycolysis in renal cancer cells by enhancing PDH activity, thereby reactivating mitochondrial function.^150^ As the only PDK inhibitor advanced to Phase II clinical trials, DCA’s clinical utility is limited by modest anticancer effects and multiple adverse reactions.^124^ Research indicates that DCA can overcome resistance to sorafenib in hepatocellular carcinoma,^151^ suggesting its potential for combination therapy with existing targeted agents in renal cancer. Multiple novel PDK inhibitors, including hordenine,^125^ JX06,^126^ and various DCA derivatives,^152^ have exhibited therapeutic effects in tumor models *in vitro*.^153^

## Targeting lactate metabolic reprogramming in ccRCC

Excessive activation of glycolytic pathways in ccRCC cells leads to substantial intracellular pyruvate accumulation. To alleviate this, ccRCC cells up-regulate LDH and MCTs, accelerating pyruvate conversion to lactate and its extrusion from cells.^154^ This metabolic reprogramming enables tumor survival in acidic environments while sustaining rapid proliferation and hypermetabolic activity. Given the critical roles of LDH and MCTs in ccRCC, they represent promising antitumor targets. Inhibiting these enzymes reduces lactate production and efflux, causing intracellular lactate accumulation, intensified acidosis, and ultimately suppressing tumor growth and survival (Table 2).

**Table 2:** Potential pharmacological targets and inhibitors targeting lactic acid metabolism.

Drug target	Mechanism of action	Small-molecule targeting agents	Research phase	Reference
LDHA	Reducte pyruvate to lactate	FX11 EGCG Galloflavin Oxamate Silibinin	Preclinical Preclinical Preclinical Preclinical Preclinical	^157^ ^158^ ^159^ ^160^ ^161^
MCTs	Transport lactic acid across membrane	7ACC1 Syrosingopine AZD3965	Preclinical Preclinical Phase I	^38^ ^162^ ^163^

Comment: The key inhibitors highlighted in this table include: (1) The MCT1 inhibitor AZD3965, which has entered Phase I clinical trials (NCT01791595), demonstrating a favorable safety profile and the ability to reverse acidification of the tumor microenvironment; (2) LDHA inhibitors such as FX11 and EGCG, which, although still in the preclinical stage, can induce oxidative stress and apoptosis, and exhibit synergistic potential with immunotherapy (e.g., anti-PD-1 antibodies). These inhibitors were selected due to their direct targeting of lactate accumulation and immune escape mechanisms in ccRCC, with AZD3965 being prioritized for immediate translational value owing to its advanced clinical progress.

Beyond its role as a metabolic waste product, lactate has recently been recognized as a key signaling molecule that drives tumor progression and therapeutic resistance through a novel post-translational modification termed lactylation.^36^ Histone lactylation, catalyzed by lactate-derived lactyl-CoA, can directly alter gene expression programs, promoting the transcription of genes involved in metastasis, immunosuppression, and notably, chemoresistance.^155^ For instance, in colorectal cancer, high levels of histone H3K18 lactylation (H3K18la) directly up-regulate the expression of the autophagy enhancer protein RUBCNL, promote autophagy, help cancer cells resist the hypoxic stress induced by bevacizumab treatment, and consequently lead to drug resistance.^156^ This reveals a direct epigenetic mechanism by which the lactate-rich tumor microenvironment fosters adaptive resistance.

## Targeting LDH

FX11, an LDHA inhibitor, increases tumor cell oxygen consumption and reactive oxygen species (ROS) production while inducing apoptosis in RCC xenograft models.^157^ Epigallocatechin-3-gallate (EGCG) suppresses LDHA and inhibits growth across multiple tumors, including RCC,^158^^,^^164^ representing a promising natural-derived antitumor agent. Both galloflavin^159^ and oxamate,^160^ validated LDHA inhibitors, inhibit tumor growth in cellular and animal studies. Remarkably, oxamate enhances pembrolizumab efficacy against non-small cell lung cancer in humanized mouse models.^165^ The natural compound silibinin also exhibits LDH inhibitory activity, effectively suppressing RCC metastasis and epithelial–mesenchymal transition (EMT) by modulating the Wnt/β-catenin signaling pathway *in vitro* and *in vivo*.^161^ Quinoline-3-sulfonamides demonstrate potent and selective LDHA inhibition with a significant reduction in intracellular lactate production, although their suboptimal pharmacokinetics limit their *in vivo* application.^166^ The bioactive flavonoid quercetin dually targets PFKFB3 and LDH, suppressing cancer glycolysis through concurrent inhibition of HK2, PFKP, and LDHA to impede proliferation.^167^ Despite the demonstrated antitumor efficacy of LDHA inhibitors in cellular and animal models, no agents targeting LDH have entered clinical trials for cancer therapy to date. Consequently, dedicated clinical research is warranted to elucidate the therapeutic potential of LDH inhibition for ccRCC treatment.

## Targeting MCTs

An *in vitro* co-culture model of renal cancer and vascular endothelial cells demonstrated that the MCT inhibitor 7ACC1 reduces lactate efflux in RCC cells, alleviates tumor acidosis, and suppresses RCC invasion, migration, and angiogenesis.^38^ This inhibition likely results from restricted lactate export causing intracellular acidosis and lactate accumulation, which feedback-inhibits upstream glycolytic flux.^38^ Metformin accelerates lactate production by enhancing glucose utilization, and its combination with MCT inhibitors exhibits synergistic tumor-suppressive effects.^168^ Syrosingopine, a dual MCT1/MCT4 inhibitor, enhanced cytotoxicity and reduced the tumor burden of multiple myeloma in a murine mode when combined with metformin.^162^ The MCT1 inhibitor AZD3965 demonstrated safety at therapeutic doses in a Phase I trial for advanced solid tumors (NCT01791595).^163^ As both isoforms MCT1/MCT4 are overexpressed in RCC, AZD3965’s limited activity against MCT4 may confer therapeutic resistance.^169^ Recent findings revealed that AZD3965 additionally inhibits *de novo* lipogenesis and enhances dendritic cell and NK cell infiltration in the tumor microenvironment.^170^ Multiple emerging MCT inhibitors (e.g., VB127,^171^ CYT-851,^172^ AR-C15558^173^) show promise but require rigorous validation in renal cancer models.

## Targeting fatty acid metabolic reprogramming in ccRCC

Targeting fatty acid metabolism reprogramming in ccRCC is an emerging therapeutic strategy. Its core lies in disrupting key nodes of lipid metabolism to impair the metabolic dependencies and survival advantages of tumor cells, thereby suppressing tumor growth and inducing cell death.

In lipogenesis, targeting rate-limiting enzymes in the *de novo* fatty acid synthesis pathway, such as ACLY, ACC, and FASN, blocks endogenous fatty acid synthesis and weakens the supply of biosynthetic substrates for tumors. For fatty acid desaturation, inhibiting SCD1 disrupts lipid membrane fluidity and increases endoplasmic reticulum stress and lipotoxicity, ultimately suppressing tumor survival. Regarding lipid uptake, suppressing the fatty acid transporter CD36 blocks tumor cells' ability to absorb exogenous fatty acids from the microenvironment, cutting off a critical nutritional supply route. In lipid catabolism, activating upstream regulators like PPARα to restore CPT1A function promotes FAO. This reverses the tumor phenotype of excessive lipid storage and insufficient oxidation, reducing lipid droplet accumulation and inhibiting tumor progression (Table 3).

**Table 3:** Potential pharmacological targets and inhibitors targeting fatty acid metabolism.

Drug target	Mechanism of action	Small-molecule targeting agents	Research phase	Reference
CD36	Transport fatty acids across membrane	SSO ABT-510 VT1021	Preclinical Phase II Phase II/Ⅲ	^174^^,^^175^ ^176^
ACLY	Produces acetyl-CoA from citrate	Cucurbitacin B ETC-1002 Morusin	Preclinical Preclinical Preclinical	^177^ ^178^ ^179^
ACC	Carboxylates acetyl-CoA to produce malonyl-CoA	TOFA ND-654 Metformin	Preclinical Preclinical Preclinical	^180^ ^181^^,^^182^ ^183^
FASN	Condenses seven malonyl-CoA and one acetyl-CoA to produce palmitate	Cerulenin C75 Orlistat TVB-2640	Preclinical Preclinical Preclinical Phase I	184, 185, 186 ^187^ 188, 189, 190 ^191^
SCD1	Desaturates palmitate to form monounsaturated fatty acids	A939572 T-3764518	Preclinical Preclinical	^192^ ^193^
CPT1	Transports long-chain fatty acids across mitochondrial membrane for fatty acid oxidation	WY-14643	Preclinical	^61^

Comment: Of the inhibitors listed in this table, the FASN inhibitor TVB-2640 is the only compound that has entered the clinical stage (Phase I), demonstrating favorable tolerability. SCD1 inhibitors such as A939572 have shown synergistic effects with mTOR inhibition in preclinical models. These inhibitors were selected based on the lipid dependency characteristic of ccRCC. TVB-2640 stands out due to its support from clinical data; however, it is important to note the limitations of single-target approaches, and future research should explore combination strategies.

## Targeting CD36

Sulfosuccinimidyl oleate sodium (SSO), a CD36 inhibitor, was employed to disrupt lipid metabolism reprogramming in oral squamous cell carcinomas (OSCCs), demonstrating dual antitumor efficacy through direct cytotoxic effects and the potentiation of antitumor immunity in both *in vitro* and *in vivo* models.^175^ An alternative strategy targets CD36 through the activation of the TSP-1-CD36-mediated apoptotic signaling pathway. Binding of CD36 expressed on tumor microvascular endothelial cells to thrombospondin-1 (TSP-1) within tumor tissue triggers programmed death of the tumor vasculature.^194^ ABT-510, a TSP-1 mimetic, underwent Phase II clinical trials in renal cancer patients. While demonstrating no significant adverse reactions, its therapeutic efficacy was inferior to sunitinib, halting further development.^174^ A separate Phase I trial combining ABT-510 with bevacizumab showed favorable tolerability and clinical activity across multiple tumor types.^195^ VT1021 currently represents the most promising CD36-targeting agent. It has exhibited positive outcomes in Phase I/II trials for ovarian cancer, pancreatic cancer, triple-negative breast cancer, and glioblastoma (NCT03364400), advancing to Phase II/III trials for glioblastoma treatment (NCT03970447). These findings indicate CD36 targeting as a viable anticancer strategy. Future research will focus on optimizing drug efficacy and safety profiles to translate this approach into clinical practice, potentially providing novel therapeutic options for cancer patients.

## Targeting ACLY

Several ACLY inhibitors have been identified, though no relevant studies exist for ccRCC. The antitumor effects of ACLY inhibitors have been investigated in other cancers. Cucurbitacin B, a natural compound belonging to the Cucurbitacin family (primary bioactive constituents in cucumbers), directly targets ACLY. It induces apoptosis in prostate cancer cells through ACLY inhibition.^177^ Bempedoic acid (ETC-1002), an approved ACLY inhibitor for hypercholesterolemia and coronary atherosclerosis,^196^^,^^197^ suppresses proliferation in breast and pancreatic cancer cell lines when combined with the CDK4/6 inhibitor palbociclib. Studies confirm that this compound promotes tumor apoptosis via ACLY blockade.^178^ Morusin, a newly discovered ACLY inhibitor, significantly reduces ACLY expression and activity in hepatocellular carcinoma cells. This triggers ROS accumulation, induces mitochondrial damage, and ultimately promotes apoptosis.^179^ Collectively, these findings underscore the translatable potential of ACLY inhibitors as multifaceted anticancer agents through metabolic disruption, apoptosis induction, and ROS-mediated cytotoxicity, highlighting an unexploited therapeutic avenue warranting dedicated exploration in ccRCC pathogenesis and treatment.

## Targeting ACC

5-Tetradecyloxy-2-furoic acid (TOFA), an early-discovered allosteric inhibitor of ACC, induces apoptosis and cell cycle arrest in RCC cells by suppressing the PI3K/Akt/mTOR pathway in preclinical studies.^180^ ND-654 and ND-646, both novel ACC inhibitors, block dimerization of the ACCα and ACCβ isoforms to inhibit enzymatic activity. Their anticancer effects have been validated in non-small cell lung cancer and hepatocellular carcinoma models,^181^^,^^182^ though preclinical studies in RCC cells remain lacking. The natural product soraphen A, another ACC inhibitor, demonstrated early anticancer potential,^198^ but is limited by poor pharmacokinetics. While most ACC inhibitors non-selectively target both ACC isoforms, ND-646 showed favorable tolerability in murine xenograft models.^181^ However, no clinical trials have been conducted on ACC inhibitors, requiring further validation of their efficacy and toxicity profiles.

The activation of AMPK to phosphorylate and inactivate ACC is an alternative ACC inhibition strategy. The antidiabetic drug metformin activates AMPK to suppress hepatic glucose utilization.^199^ It inhibits RCC cells *in vitro* and *in vivo*,^183^ with reported antitumor effects across multiple cancers.^200^^,^^201^ However, the anticancer mechanism of metformin remains complex: studies confirm its action via mTOR pathway inhibition,^4^^,^^202^ yet whether ACC suppression contributes to antitumor effects requires further investigation. Clinical efficacy is contentious, while retrospective metastatic RCC studies indicate the metformin and sunitinib combination therapy improves survival outcomes and potentially extends OS.^203^^,^^204^ A real-world analysis demonstrated that statin use significantly prolonged OS (HR 0.48, 95% CI 0.26–0.87; *P* = 0.016), whereas metformin showed no prognostic correlation,^205^ underscoring the imperative for rigorous validation of metformin’s therapeutic role in ccRCC.

## Targeting FASN

Cerulenin, an early-discovered FASN inhibitor, has demonstrated anticancer effects across multiple tumor cell lines.^184^, ^185^, ^186^ Its derivative C75 reduces cellular viability and growth, diminishes migration, and enhances apoptosis via cell cycle arrest in various ccRCC cell lines.^187^ However, both agents induce appetite suppression and weight loss in murine models, potentially linked to hypothalamic malonyl-CoA accumulation resulting from FASN inhibition,^206^ posing clinical risks for exacerbating cancer cachexia. The extensively studied FASN inhibitor Orlistat, an FDA-approved drug for obesity management,^207^ exhibits potent antitumor activity in preclinical models.^208^ When combined with chemotherapeutic agents, Orlistat significantly enhances therapeutic efficacy without observable adverse reactions.^188^, ^189^, ^190^ Nonetheless, its poor solubility and oral bioavailability limit systemic anticancer applications.^209^ Distinguishably, TVB-2640, the only FASN inhibitor advanced to clinical trials, showed favorable tolerability in Phase I studies with no dose-limiting gastrointestinal or serochemical toxicities,^191^ meriting dedicated investigation into its therapeutic potential for ccRCC.

## Targeting SCD1

A939572, an SCD1 inhibitor, significantly suppresses proliferation and promotes apoptosis in ccRCC cells *in vitro*, potentially through SCD1 inhibition-induced endoplasmic reticulum stress.^55^^,^^192^ This study also revealed synergistic interplay between SCD1 and the mTOR pathway: combining A939572 with temsirolimus markedly enhanced antitumor efficacy compared to monotherapy, while no synergy occurred with tyrosine kinase inhibitors (TKIs).^55^ A939572 exhibits similar antiproliferative and pro-apoptotic effects across diverse tumor type.^210^, ^211^, ^212^ However, murine models have demonstrated adverse effects, including xerophthalmia, alopecia, and xeroderma.^213^ The novel SCD1 inhibitor T-3764518 reduced tumor volume without significant toxicity in ccRCC xenograft models.^193^ Other SCD1 inhibitors, such as SSI-4,^214^ agrimonolide,^215^ Aramchol^216^ and MF-438,^217^ have demonstrated antitumor efficacy in non-renal cancers but require validation in RCC cell lines. Notably, no SCD1 inhibitors have entered oncology clinical trials. Among these SCD1 inhibitors, only Aramchol reached Phase II trials (NCT02279524) for nonalcoholic steatohepatitis, showing no serious adverse events at 400 mg or 600 mg dosing.^216^ Given the compelling preclinical antitumor profiles of SCD1 inhibitors, dedicated clinical exploration is warranted.

## Targeting CPT1A

Therapeutically, diminished CPT1A expression in ccRCC cells renders CPT1A inhibition therapeutically ineffective. No direct CPT1A activators have been identified to date. An alternative approach involves activating peroxisome proliferator-activated receptor alpha (PPARα), a pivotal transcriptional regulator of lipid metabolism. WY-14643, by activating PPARα, induces carnitine palmitoyltransferase 1A (CPT1A) expression to augment fatty acid metabolism.^61^ This discovery establishes a novel therapeutic axis for RCC, warranting dedicated exploration of PPARα agonists for ccRCC treatment with rigorous validation of their safety and efficacy profiles.

## Targeting OXCT1

Although there are currently no drugs targeting ketone body metabolic enzymes for ccRCC, exploring the functional mechanisms of ketone body metabolism-related enzymes in ccRCC holds significant value given its unique lipid metabolic characteristics. Current studies have found that the OXCT1 inhibitor acetohydroxamic acid can inhibit ketone body metabolism, promote the accumulation of ketone bodies, suppress the growth of liver tumors in mice, and significantly enhance the efficacy of lenvatinib.^218^ It may offer a novel synergistic strategy for treating ccRCC.

## Targeting amino acid metabolic reprogramming therapy in ccRCC

In ccRCC, profound amino acid metabolic reprogramming not only provides survival advantages for tumor cells but also shapes an immunosuppressive tumor microenvironment. Notably, metabolic alterations in glutamine, tryptophan, and arginine are particularly significant, with these processes being driven by specific key enzymes. Consequently, targeting these metabolic enzymes to reverse the cancer-promoting metabolic state has emerged as a promising therapeutic avenue for ccRCC. This section provides a detailed discussion on these aspects (Table 4).

**Table 4:** Potential pharmacological targets and inhibitors targeting amino acid metabolism.

Drug target	Mechanism of action	Small-molecule targeting agents	Research phase	Reference
SLC1A5	Transport glutamine across membrane	V9302 KM8094	Preclinical Preclinical	^76^ ^219^
GLS1	Produces acetyl-CoA from citrate	CB-839	Phase II	^220^
IDO1 TDO IDO1&TDO	Produces kynurenine from tryptophan Produces kynurenine from tryptophan Produces kynurenine from tryptophan	Epacadostat LM10 M4112	Phase Ⅲ Preclinical Phase I	^221^ ^222^ ^223^

Comment: Of the inhibitors listed in this table, the GLS1 inhibitor Telaglenastat (CB-839) has progressed to Phase II trials (e.g., the ENTRATA trial), demonstrating potential when combined with mTOR inhibitors. Although the IDO1 inhibitor Epacadostat did not meet expectations in Phase III trials, it highlighted the significance of metabolic-immune crosstalk. These inhibitors were selected for their targeting of ccRCC’s glutamine addiction and immunosuppressive microenvironment. Telaglenastat, being the most clinically advanced, warrants prioritized attention for further investigation.

## Targeting metabolic enzymes in glutamine metabolism

The aberrant dependence on and addiction to glutamine in ccRCC represent a critical metabolic vulnerability and potential therapeutic target. This glutamine addiction stems from frequent VHL gene loss-of-function mutations and consequent hyperactivation of the HIF pathway, which drives tumor cells to utilize glutamine as a primary carbon source, nitrogen donor, and energy substrate for biosynthesis, redox homeostasis maintenance, and support of rapid proliferation and metastatic progression. Consequently, targeting glutamine uptake, transport, and key metabolic enzymes (e.g., GLS1) to disrupt metabolic flux constitutes a highly promising intervention strategy (Table 4).

### Targeting SLC1A5

Current research on SLC1A5 inhibitors remains confined to the preclinical stage. The SLC1A5 inhibitor V-9302 significantly suppresses proliferation, invasion, and migration in human ccRCC A498 and Caki1 cell lines. *In vivo* studies further demonstrated the potent inhibition of xenograft tumor growth, though accompanied by significant weight loss in murine models.^76^ The monoclonal antibody KM8094, targeting SLC1A5, impedes glutamine uptake in tumor cells. It inhibits gastric cancer xenograft growth *in vivo* and exhibits synergistic effects with docetaxel.^219^ Targeted inhibition of SLC1A5-mediated glutamine transport robustly suppresses cancer cell proliferation, survival, and tumorigenesis both *in vitro* and *in vivo*. With emerging novel SLC1A5 inhibitors, this approach holds promise for pioneering new therapeutic avenues in renal carcinoma.^224^

### Targeting GLS1

Telaglenastat (CB-839), a GLS inhibitor, reduces tumor glutamine utilization. When combined with everolimus in RCC cells, it decreases glucose and glutamine consumption while exhibiting synergistic anti-proliferative effects.^79^ Phase I trials demonstrated favorable safety profiles for telaglenastat monotherapy and combinations with cabozantinib or everolimus.^225^^,^^226^ However, the CANTATA study revealed that telaglenastat plus cabozantinib failed to improve median progression-free survival (PFS) in advanced RCC patients (HR 0.94; 95% CI 0.74–1.21; *P* = 0.65). Similarly, its combination with nivolumab showed good tolerability but no significant efficacy difference in clinical trials.^227^ Notably, the Phase II ENTRATA trial demonstrated a shorter median PFS for the everolimus plus telaglenastat group (HR 0.64; 95% CI 0.34–1.20; *P* = 0.079).^220^ These mixed outcomes indicate the potential yet unresolved therapeutic value for GLS inhibitors combined with mTOR inhibitors in advanced RCC.

## Targeting metabolic enzymes in tryptophan metabolism

Dysregulated tryptophan metabolism, mediated by key enzymes such as IDO and TDO, contributes significantly to immunosuppression in ccRCC by depleting tryptophan and generating kynurenine-based AhR ligands. Targeting these enzymes represents a promising strategy to reverse immune evasion and enhance the response to immunotherapy. The following section reviews recent advances in the development of IDO1 and TDO inhibitors.

### Targeting IDO1

The critical role of IDO1 in tumorigenesis and development has stimulated the development and extensive exploration of inhibitors targeting this pathway. Among them, the selective IDO1 inhibitor Epacadostat, by inhibiting the key enzyme in the tryptophan metabolic pathway, is mechanistically believed to reverse the immunosuppressive state of the tumor microenvironment, thereby enhancing the anti-tumor activity of PD-1 inhibitors. However, the results from the KEYNOTE-679/ECHO-302 Phase III clinical study showed that in the first-line treatment of metastatic renal cell carcinoma (mRCC), pembrolizumab combined with Epacadostat (64 patients), compared with standard targeted therapy with sunitinib/pazopanib (65 patients), after a median follow-up of 10.3 months, yielded similar objective response rates (ORRs) between the two groups (combination group: 31.3% [95% CI: 20.2–44.1] *vs.* targeted therapy group: 29.2% [95% CI: 18.6–41.8]).^221^ This finding is consistent with the results of several other Phase III clinical trials (including NCT03260894, NCT03322540, NCT03322566, NCT03358472, NCT03361865, NCT03374488, etc.). Although preclinical studies demonstrated a theoretical prospect of enhancing antitumor immunity by modulating tryptophan metabolism, these clinical studies failed to confirm the expected therapeutic advantage of combining the IDO1 inhibitor epacadostat with PD-1 inhibitors across multiple cancer types.^228^ Three Phase III clinical trials (NCT03329846, NCT03386838, NCT03417037) evaluating the IDO1 inhibitor BMS-986205 in combination with nivolumab (an anti-PD-1 antibody) have been terminated. This outcome is consistent with the findings of multiple previous similar studies, confirming that BMS-986205 failed to significantly enhance the clinical efficacy of PD-1 inhibitors. These results further indicate that the IDO1 inhibition strategy has not achieved its expected therapeutic value in clinical translation. The clinical efficacy of IDO1 inhibitors is limited primarily by two factors: first, existing drugs can only inhibit the enzymatic activity of IDO1 but fail to block its non-enzymatic functions (such as signal transduction), which may allow immune escape to persist^229^; second, the inhibitory effect *in vivo* is incomplete, as patients' blood kynurenine levels remain relatively high, potentially due to compensatory mechanisms involving other metabolic enzymes like TDO. Therefore, developing novel inhibitors capable of simultaneously suppressing both the enzymatic activity and non-enzymatic functions of IDO1, as well as designing dual-targeting IDO/TDO drugs to overcome metabolic compensation effects, may represent crucial research directions for breaking through the current therapeutic bottlenecks.

### Targeting TDO

Although the research scale of TDO inhibitors is not as extensive as that of IDO1 inhibitors, it has demonstrated immunomodulatory potential. In studies on oral squamous cell carcinoma (OSCC), the TDO inhibitor LM10 effectively enhanced T cell anti-tumor activity and alleviated their suppressed state by reversing the conversion of CD4+ T cells into Tregs and restoring the function of exhausted CD8+ T cells.^222^ In a study on small cell lung cancer, inhibition of IDO1 led to a compensatory upregulation of TDO expression, whereas the use of the dual IDO1/TDO inhibitor AT-0174 significantly suppressed tumor growth.^91^ These results indicate that simultaneously targeting both IDO1 and TDO can effectively overcome the metabolic compensation mechanism triggered by single-pathway inhibition, thereby enhancing antitumor efficacy. However, no relevant preclinical studies on TDO inhibitors or IDO1/TDO dual inhibitors have been conducted in the context of ccRCC. The IDO1/TDO dual inhibitor M4112 was shown to be safe in a Phase I clinical trial but was terminated early due to its failure to reduce serum kynurenine levels (NCT03306420).^223^ Other IDO1/TDO dual inhibitors, such as LPM3480226 (NCT03844438) and DN1406131 (NCT03641794), have demonstrated antitumor effects in preclinical studies and are currently undergoing Phase I clinical trials to evaluate their safety profiles. Future research should focus on developing IDO1/TDO dual inhibitors and combining them with immune checkpoint inhibitors, aiming to overcome metabolic compensation, enhance antitumor immune responses, and provide new therapeutic strategies for patients with RCC.

## Targeting arginine metabolism

Current research focuses on leveraging arginine deprivation therapy, which targets the arginine auxotrophy of tumor cells to deplete arginine in the microenvironment, thereby selectively inducing tumor cell death and offering a novel strategy for precision cancer treatment. As a core agent of this approach, the pegylated arginine deiminase ADI-PEG20 efficiently degrades circulating arginine and has demonstrated favorable safety and preliminary efficacy in multiple clinical trials.^230^^,^^231^ Although the existing data are encouraging, research on ADI-PEG20 in ccRCC remains a gap, warranting further exploration in this field to validate its therapeutic potential.

## Conclusions

ccRCC, a classic example of a metabolism-driven malignant tumor, is characterized by aberrant activation of the VHL-HIF signaling axis, which leads to extensive metabolic reprogramming. This reprogramming not only directly drives tumor proliferation by enhancing the Warburg effect, promoting lipid accumulation, and altering amino acid metabolism but also indirectly contributes to resistance to conventional targeted therapies and immunotherapies by shaping an immunosuppressive tumor microenvironment. Although immunotherapy has achieved significant breakthroughs in recent years,^232^^,^^233^ its efficacy is often limited by the immunosuppressive microenvironment dominated by metabolic alterations. For instance, lactate produced by excessive glycolysis is extruded via monocarboxylate transporters (MCTs), leading to local tumor acidosis that directly suppresses CD8+ T cell function and promotes M2 macrophage polarization. Meanwhile, down-regulation of ketone body metabolism enzymes (e.g., HMGCS2) may impair the metabolic adaptability of immune cells. Consequently, targeting key metabolic nodes (such as the LDHA inhibitor FX11 or the MCT inhibitor AZD3965) has shown potential in preclinical studies to reverse immunosuppression and enhance the efficacy of PD-1/PD-L1 inhibitors. However, metabolic intervention carries inherent complexities and risks. For example, while targeting PFKFB3 can inhibit glycolysis, it may inadvertently enhance tumor invasiveness due to metabolic plasticity.^234^ Additionally, compensatory pathway activation (e.g., up-regulation of glutaminolysis upon glycolysis inhibition) often limits the efficacy of monotherapies. Future strategies must focus on multi-dimensional innovations: at the therapeutic level, prioritizing exploration of combination therapies (such as expanding clinical trials of metabolic inhibitors with immune checkpoint inhibitors and optimizing treatment sequences to balance efficacy and toxicity); at the drug development level, designing multi-target inhibitors (e.g., dual-targeting IDO1/TDO molecules) to prevent metabolic compensation; at the technological level, integrating patient-derived organoid models and spatial metabolomics technologies to decipher intra-tumoral metabolic heterogeneity and guide personalized therapy; and simultaneously validating non-invasive biomarkers for dynamic therapy response monitoring. Ultimately, deep collaboration among metabolic biologists, immunologists, and clinical oncologists will propel targeting ccRCC metabolic reprogramming from mechanistic research into clinical practice, offering a new paradigm for overcoming therapy resistance.

## Literature search strategy

To comprehensively cover the latest research advances in metabolic reprogramming and targeted therapy for clear cell renal cell carcinoma (ccRCC), a systematic literature search was conducted. The search was performed in electronic databases, including PubMed, Web of Science Core Collection, and Scopus. The time range was from the inception of each database to August 2025. The following core search terms and their combinations were used: (“clear cell renal cell carcinoma” OR ccRCC) AND (“metabolic reprogramming” OR “Warburg effect” OR glycolysis OR “fatty acid metabolism” OR “glutaminolysis”) AND (“targeted therapy” OR inhibitor OR “clinical trial”). The search was restricted to English-language publications of original research articles and reviews. The initial search results were screened by reading titles and abstracts to exclude irrelevant studies (e.g., non-ccRCC cancers, non-metabolism-related research). Ultimately, a total of over 200 relevant publications were included in this comprehensive review.

## CRediT authorship contribution statement

**Jia-tao Yao:** Writing – original draft. **Peng-cheng Hu:** Writing – original draft. **Xiao-wei Li:** Writing – original draft. **Jia-cheng Xu:** Writing – review & editing. **Ke-jie Wang:** Writing – review & editing. **Sha-zhou Ye:** Writing – review & editing. **Xiang-yu Meng:** Writing – review & editing. **Hai-chao Chen:** Project administration, Funding acquisition. **Yu Liang:** Project administration, Funding acquisition. **Qi Ma:** Supervision, Project administration, Funding acquisition.

## Funding

This work was supported by Ningbo Clinical Research Center for Urological Disease (China) (No. 2019A21001); Ningbo Top Medical and Health Research Program (China) (No. 2022020203); Zhejiang Engineering Research Center of Innovative technologies and diagnostic and therapeutic equipment for urinary system diseases; Zhejiang Provincial Medical and Health Science and Technology Program (China) (No. 2025KY1317); 10.13039/100007834Ningbo Natural Science Foundation (China) (No. 2024J342).

## Conflict of interests

The authors declare no potential conflict of interests related to this work.
